# Effect of Different Light Intensities on Total Phenolics and Flavonoids Synthesis and Anti-oxidant Activities in Young Ginger Varieties (*Zingiber officinale* Roscoe)

**DOI:** 10.3390/ijms11103885

**Published:** 2010-10-12

**Authors:** Ali Ghasemzadeh, Hawa Z. E. Jaafar, Asmah Rahmat, Puteri Edaroyati Megat Wahab, Mohd Ridzwan Abd Halim

**Affiliations:** 1 Department of Crop Science, Faculty of Agriculture, University Putra Malaysia, 43400 UPM Serdang, Selangor, Malaysia; E-Mails: upmali@yahoo.com (A.L.); putri@agri.upm.edu.my (P.E.M.W.); ridzwan@agri.upm.edu.my (M.R.A.H.); 2 Department of Nutrition & Dietetics, Faculty of Medicine & Health Sciences, University Putra Malaysia, 43400 UPM, Serdang, Selangor, Malaysia; E-Mail: asmah@medic.upm.edu.my

**Keywords:** TP, TF, light intensity, Halia Bara, Halia Bentong, DPPH

## Abstract

Nowadays, phytochemicals and antioxidants in plants are raising interest in consumers for their roles in the maintenance of human health. Phenolics and flavonoids are known for their health-promoting properties due to protective effects against cardiovascular disease, cancers and other disease. Ginger (*Zingiber officinale*) is one of the traditional folk medicinal plants and it is widely used in cooking in Malaysia. In this study, four levels of glasshouse light intensities (310, 460, 630 and 790 μmol m^−2^s^−1^) were used in order to consider the effect of light intensity on the production, accumulation and partitioning of total phenolics (TP), total flavonoids (TF) and antioxidant activities in two varieties of Malaysian young ginger (*Zingiber officinale*). TF biosynthesis was highest in the Halia Bara variety under 310 μmol m^−2^s^−1^ and TP was high in this variety under a light intensity of 790 μmol m^−2^s^−1^. The highest amount of these components accumulated in the leaves and after that in the rhizomes. Also, antioxidant activities determined by the 1,1-Diphenyl-2-picryl-hydrazyl (DPPH) assay in both of varieties, increased significantly (p ≤ 0.01) with increasing TF concentration, and high antioxidant activity was observed in the leaves of Halia Bara grown under 310 μmol m^−2^s^−1^. The ferric reducing (FRAP) activity of the rhizomes was higher than that of the leaves in 310 μmol m^−2^s^−1^ of sun light. This study indicates the ability of different light intensities to enhance the medicinal components and antioxidant activities of the leaves and young rhizomes of *Zingiber officinale* varieties. Additionally, this study also validated their medicinal potential based on TF and TP contents.

## 1. Introduction

Ginger (*Zingiber officinale* Roscoe) is a famous and widely used herb, especially in Asia, which contains several interesting bioactive constituents and possesses health promoting properties. Rhizomes of ginger plants (family Zingiberaceae) have been widely used as spices or condiments [1]. Rhizomes are eaten raw or cooked as vegetables and used for flavoring food in Malaysia. The major commercially cultivated species in Asia. *Zingiber officinale*, *Curcuma longa*, and *Alpinia galangal*, are used for traditional medicine and food flavoring. As traditional medicine, rhizomes of ginger plants are consumed by women during illness and confinement. Rhizome extract is also taken as a carminative for relieving flatulence [2]. In Malaysia, only ginger rhizomes are consumed as food flavoring and the leaves are throwing away. In Thailand, leaves of ginger are eaten as salad and boiled leaves of *Hedychium coronarium* are applied to relieve stiff and sore joints.

Plants are potential sources of natural bioactive compounds such as secondary metabolites and antioxidants. They absorb the sun light and produce high levels of oxygen and secondary metabolites by photosynthesis. Medicinal components produced are stored in plant leaves. Most of the secondary metabolites of herbs and spices are commercially important and find use in a number of pharmaceutical compounds. Flavonoids and phenolics are most important groups of secondary metabolites and bioactive compounds in plants [3]. They are also a kind of natural product and antioxidant substance capable of scavenging free superoxide radicals, anti-aging and reducing the risk of cancer. It was found that flavonoids function to reduce blood-lipid and glucose and to enhance human immunity [4]. Flavonoids have important roles in human life and health. Their function in human health is supported by the ability of flavonoids to induce human protective enzyme systems, and by number of epidemiological studies suggesting protective effects against cardiovascular diseases, cancers and other related diseases [5]. Previous studies suggested that some flavonoids such as catechin and quercetin could be able to control cancer cell growth in the human body [6–8]. Shuklah *et al.* [8] reported cancer preventive properties of ginger. He believed that ginger extract is able to control or inhibit cancer cell growth. This ability is related to flavonoid and polyphenolic components of fresh ginger extract. At present, flavonoids are extracted from ginkgo leaves [9], kudzu root [10], lotus leaves [11] and ginger rhizomes [12]. Flavonoids also had a positive effect on alleviating malaise such as glycuresis, hyperlipemia and hypertension. However, the flavonoid content of some materials is limited and other materials are mainly of dietary or medicinal use; none of these materials can be produced on a large enough scale to meet the demand for flavonoids.

Ginger is known as a resource with higher phenolic components, that has a wide source and low price [13,14], and therefore it can serve as a cheap and important material in food.

Phenolics and flavonoids synthesis in plants is influenced by environmental factors, plant age, leaf maturity, *etc* [15]. Light is known to regulate not only plant growth and development, but also the biosynthesis of both primary and secondary metabolites [15,16]. Phenolic biosynthesis requires light or is enhanced by light, and flavonoid formation is absolutely light dependent and its biosynthetic rate is related to light intensity and density [17]. However, different plants had a different response to light intensity alteration and the resulting total flavonoids (TF) and total phenolics (TP) production. Previous studies showed that changes in light intensity were able to change the production of TF and TP in herbs [18]. According to Briskin *et al.* [19] and Kurata *et al.* [20], light intensity—with changes in plant morphology and physiological characteristics—affected the medicinal component (TP) in herbs. According to previous studies, a positive and significant relationship has been observed between TP, TF and antioxidant activities in plants [19,20]. It seems that different light intensities had a direct effect on antioxidant activities in plants with increasing TP and TF content. It is well known that phenolic compound and flavonoids are one of the major contributors to the antioxidant activity of herbs and medicine plants [21,22]. It was suggested that increasing phenolic and flavonoid components in shaded plants is related to lower temperatures under this condition. High temperature increases anthocyanin degradation in grape skin, together with a decrease in expression of flavonoid biosynthetic [23]. On the other hand, low temperature increases anthocyanin production, as has been observed in plants [24,25].

Information on the flavonoid contents of plant foods and plant parts commonly consumed in Malaysia are still scarce. Such data is useful to provide information on foods containing high levels of these beneficial components. Also, there is no information about the effects of differing light intensity on TP and TF biosynthesis and antioxidant properties of leaves and rhizomes of ginger species. In the present study, we comprehensively and systematically compared the total flavonoids and phenolic contents of leaves, stems and rhizomes of ginger species under different light intensities. The main objective of the current study was to maximize the secondary metabolite production, including medicine components (TF and TP), of a Malaysian ginger variety (Halia Bentong) under a controlled environment.

## 2. Results and Discussion

### 2.1. Total Phenolics (TP) Content

The result obtained from the preliminary analysis of TP is shown in Table 1. It is apparent that TP accumulation and partitioning in the plant was considerably affected by the differing light intensity. The different light intensities had a significant (p ≤ 0.01) effect on the TP produced (Table 1). The results show that in both varieties of ginger, the leaves had a higher TP content (33 mg/g dry weight in Halia Bentong and 39.06 mg/g dry weigh in Halia Bara) under a light intensity of 790 μmol m^−2^s^−1^ compared with other light intensities. The partitioning of TP at 16 weeks after planting was: leaves > rhizomes > stems. The results obtained from the preliminary analysis of TF and TP are shown in Table 1. According to the results in this table, factors (light, varieties and plant parts) had a significant effect (p ≤ 0.01) on TP accumulation and partitioning in both varieties. On the other hand, different light intensity had a significant effect on the production, accumulation and partitioning in different part of plants and also different varieties had a different concentration of TP in different part of plants. TF and TP content in ginger grown in a glasshouse under normal light intensity (790 μmol m^−2^s^−1^) were reported in a previous study by present authors [12] and the results from this light intensity (790 μmol m^−2^s^−1^) is shown in Table 1 as a standard condition to compare with the results of lower light intensities.

Ginger is a semi shade plant, and the results of this study showed that 790 μmol m^−2^s^−1^ is suitable light intensity for maximum TP production. The increased TF in Halia Bentong with increasing shade could be associated with significantly higher leaf chlorophyll and carotenoid contents under lower light levels. TP levels were higher in leaves of both of varieties under a light intensity of 790 μmol m^−2^s^−1^. Contrary to our results, higher phenolic contents and antioxidant activity have been reported in rhizomes than in leaves of *Z. officinale* [26]. These studies involved one or two ginger species and it is not known whether their comparisons were based on plant samples from the same or different locations. However, the results of Chan *et al.* [27] supported our findings. High levels of TP was reported in ginger leaves when compared with rhizomes in *Zingiber officinale* [27]. The quality and yield of ginger rhizomes increased when grown in the shade because nutrient uptake increased [28].

Increases in soluble phenolics, such as the intermediates in lignin biosynthesis, can reflect the typical anatomical change induced by stressors: an increase in cell wall endurance and the creation of physical barriers preventing calls against harmful action [29]. However, some products in plants such as anthocyanin, cumarin and lignin are biosynthesized in the course of phenolic compounds being transformed into flavonoids [30]. TP content in both varieties when compared to other medicine plants, for example Calotropis (stems: 14.97 mg/g DW; leaves 14.94 mg/g DW), Hibiscus (stems: 7.04 mg/g DW; leaves 29.96 mg/g DW), Parthenium (stems: 4.88 mg/g DW; leaves 8.29 mg/g DW) and Kigelia (stems: 33.4 mg/g DW; leaves 15.04 mg/g DW) [31], showed good potential in different parts of the plant.

### 2.2. Total Flavonoids (TF) Content

It is apparent from Table 1 that TF accumulation and partitioning was considerably affected by the different light intensities. Differing light intensity also had a significant (p ≤ 0.01) effect on the TP produced (Table 1). The results show that leaves in both of varieties have a higher content of TF (5.95 mg/g dry weight in Halia Bentong and 8.45 mg/g dry weight in Halia Bara) under 310 μmol m^−2^s^−1^ of light intensity. A similar trend of increasing TF content with decreasing light intensity were seen in *Tanacetum parthenium* and strawberry [32,33] and in some medicinal plants illustrating a considerable influence of low irradiance on enhancement of plant TF [19]. Michel *et al.* [34] reported TF production related to plant pigments (chlorophyll and carotenoids), and in contrast to flavonoids, the xanthophyll cycle seems to be mainly relevant to the protection of photosynthesis against sudden increases in light intensity. Chan *et al.* [27] reported much greater concentrations of flavones and flavonols in leaves of vegetables that are exposed to shade. This finding is in agreement with Bergquist’s findings [35], which showed that the use of shade netting is acceptable for production of baby spinach in regard to flavonoid concentration and composition.

From Table 1, it is apparent that TF partitioning to the leaves, stems and rhizomes increased steadily and substantially with decreasing light intensity to 310 μmol m^−2^s^−1^. With decreasing TP in the leaves and rhizomes from 790 to 310 μmol m^−2^s^−1^, TF content increased significantly in the leaves and rhizome of both varieties. According to the shikmic acid pathway, phenolic compounds are produced first and after that phenolic acids (elagic acid, tannic acid and vanillin), hydroxy cinnamic acid (caffeic acid, ferulic acid, curcumin, cournarins), lignas and flavonoids produced from phenolic compounds in other cycles [36]. Therefore, according to Table 1 data, it can be hypothesized that the biosynthesis of TF from phenolic compounds in plants grown under light intensity of 310 μmol m^−2^s^−1^ was more than other phenolic components (phenolic acid, hydroxyl cinnamic and lignas) and low levels of TF in plants grown under 790 μmol m^−2^s^−1^ of light intensity is related to more biosynthesis of other phenolic components compared with flavonoids. It can be seen from the data in Table 1 that the TF content—when compared with other plant for example onion leaves (2.72 mg/g DW), bird chili (1.66 mg/g DW), papaya shoots (1.26 mg/g DW), guava (1.12 mg/g DW), semambu leaves (2.04 mg/g DW) and black tea (1.49 mg/g DW) [37]—shows high level in Halia Bara leaves and rhizomes when grown under 310 μmol m^−2^s^−1^ of light intensity. The results of our study are consistent with other studies and suggest that high content of some phenolic components such as cinamic acid can inhibit flavonoid biosynthesis with inhibition of PAL (phenylalanine ammonia lyase) enzyme activity [38].

The synthesis of isoflavones and some other flavonoids is induced when plants are infected or injured [27,28], or under low light and low nutrient conditions [39,40].

### 2.3. Antioxidant Activity

#### 2.3.1. 1,1-Diphenyl-2-picryl-hydrazyl (DPPH) Assay

DPPH is one of the famous free radicals and is usually used for testing preliminary radical scavenging activity of a compound or plant extract. The free radical scavenging property may be one of the mechanisms by which this drug is effective in traditional medicine. Most of the tannins and flavonoids are phenolic components and may be responsible for the antioxidant properties of many plants [41,42]. There are no studies comparing the antioxidant properties of leaves and rhizomes of ginger species grown under different light intensities.

**Figure 1:**
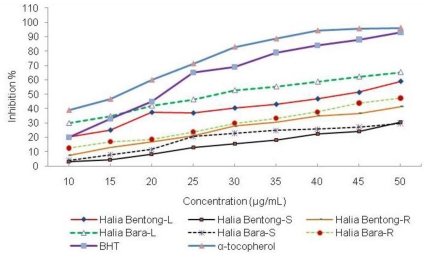
DPPH radical scavenging activity of the methanolic extracts in different parts of the two varieties of *Zingiber officinale* plants grown under 310 μmol m^−2^s^−1^ light intensity compared with positive controls, BHT and α-tocopherol. L, S and R, respectively are: leaves, stems and rhizomes of ginger. All analyses were mean of triplicate measurements.

Two different light intensities (310 and 790 μmol m^−2^s^−1^), based on the high content of TF and TP, were chosen for the DPPH scavenging assay. The results of the assay are shown in Table 2 and Figure 1. From the data in Figure 1 and Table 2, it is apparent that the extracts from ginger grown under 310 μmol m^−2^s^−1^ showed a significant effect in inhibiting DPPH, reaching up to 60% in Halia Bara leaves and 55% in Halia Bentong leaves at concentration 45 μg/mL and its IC_50_ was 27 μg/mL for Halia Bara and 40 μg/mL for Halia Bentong Leaves. However, at the same concentration (27 and 40 μg/mL), the IC_50_ values of Halia Bentong and Halia Bara leaves grown under 790 μmol m^−2^s^−1^ were 42 and 40%, respectively.

Antioxidant activities were higher in the leaves compared to stems and rhizomes under 310 μmol m^−2^s^−1^. With increasing TF accumulation, the free radical scavenging power increased in ginger. Antioxidant activities decreased in samples from all parts of the plant with increasing light intensity. According to Table 1 and 2, DPPH scavenging activities in plants which grown under 310 μmol m^−2^s^−1^ and containing high levels of TF was more than from plants which were grown under 790 μmol m^−2^s^−1^ with high levels of TP. On the other hand, it seems that the role of TF in ginger is more important than TP for scavenging of free radicals.

The free radical scavenging activities in ginger grown under normal light intensity (790 μmol m^−2^s^−1^) was reported in a previous study by the present authors [12]. The present findings seem to be consistent with other research. Essential oils extracted from leaves of *Aframomum giganteum* had higher antioxidant activity in comparison with those extracted from rhizomes [43]. Leaves of *A. zerumbet* showed higher inhibition of b-carotene oxidation and radical scavenging activity than did rhizomes [44]. Antioxidants are secondary metabolites produced by most plants but in different contents to protect against oxidative damage by free radicals [45]. In the family Zingiberaceae and especially *Zingiber officinale*, it is generally believed that antioxidants produced by the plant are transported to the rhizomes where they accumulate [46]. This may imply that rhizomes would have higher antioxidant activity than other plant parts. However, the results of this study showed that this might not be true, as the majority of the species studied had significantly higher flavonoids contents and antioxidant activity in leaves than in rhizomes. Similar observations have been made by Chan *et al.* [27], who reported that the leaves of ginger with a high level of TF had high antioxidant activities in comparison with rhizomes. According to TF and TP content in leaves, the high antioxidant activities in leaves maybe able to highlight the relationship between them. These results may explain the relatively good correlation between TF and free radical scavenging in ginger leaves and rhizomes. This relationship was also reported in previous studies [47,48]. Further work is required to establish the components in phenolics and flavonoids that may contribute to the high antioxidant activities so far observed.

#### 2.3.2. Reducing Ability, Ferric Reducing Antioxidant Potential (FRAP)

Several methods are known to measure the total antioxidant capacity of herbs, including FRAP assay, which we have adopted in this study. The FRAP assay depends upon the reduction of ferric tripyridyltriazine (Fe(III)-TPTZ) complex to the ferrous tripyridyltriazine (Fe(II)-TPTZ) by a reductant at low pH.

It can be seen from the data in Table 3 that the ferric reducing antioxidant potential of ginger varieties increased with decreasing light intensity. The FRAP values for the methanolic extracts of the leaves, rhizomes and stems of both varieties were significantly lower than those of vitamin C (3107.28 μmol Fe(II)/g) and α-tocopherol (953 μmol Fe(II)/g), but higher than that of BHT (74.31 μmol Fe(II)/g). Significant differences (p ≤ 0.01) were observed between different light intensities, varieties and different parts of the plant for ferric reducing (Table 3). The ferric reducing ability (FRAP assay) is widely used to evaluate the antioxidant component in dietary polyphenols [49].

The antioxidant activity of rhizomes in these varieties of *Zingiber officinale* was significantly enhanced when grown under 310 μmol m^−2^s^−1^. According to the TF and TP results, under this light intensity, synthesis of TF increased but synthesis of TP decreased. Therefore, increasing antioxidant activity in young ginger rhizomes may be related to increasing TF accumulation. These findings further support the idea of a positive relationship between phenolic compounds and flavonoids with antioxidant activities of plants. However antioxidant activity is found to be linearly proportional with phenolic compounds and especially flavonoids. Oktay *et al.* [49] and Ghasemzadeh *et al*. [12] reported strong positive relationships between total flavonoids contents and antioxidant activity, which appears to be the trend in many plant species.

## 3. Experimental Section

### 3.1. Plant Material and Maintenance

Rhizomes of Halia Bentong and Halia Bara (*Zingiber officinale)* was germinated for two weeks in small pots and then transferred to white polyethylene bags which were to be filled with soilless mixture including burnt rice husk and coco peat (ratio 1:1). Ginger is a semi-shade loving plant and needs shade for growth and rhizome production. The plants were grown under four level of glasshouse shade (0%, 20%, 40% and 60% shade) at the glasshouse complex of the University Putra Malaysia (UPM). The average light intensity passing through in each shading treatment was 790, 630, 460 and 310 μmol m^−2^s^−1^ of photosynthetically active radiation (PAR), respectively. The experiment was factorial based on a Randomized Complete Block with 3 replications. Plants were harvested 16 weeks after planting and total flavonoids, total phenolics and antioxidant activities in different parts of the plants were measured. The location of the experiment was at the glasshouse complex of the University Putra Malaysia (UPM).

### 3.2. Extract Preparation

Leaves, stems and rhizomes were freeze dried to constant weights before being used in the extraction. For antioxidant analysis, the leaves, stems and rhizomes were made into powder and one gram of the powder was used in the extraction using methanol (50 mL). The solution was then swirled for 1 h at room temperature using an orbital shaker. Extracts were filtered under suction and stored at −20 °C for further use.

### 3.3. Determination of Total Phenolic Contents (TP)

The total phenolic content was determined using Folin–Ciocalteu reagents with analytical grade gallic acid as the standard. 1 mL of extract or standard solution (0–500 mg/L) was added to deionized water (10 mL) and Folin-Ciocalteu phenol reagents (1.0 mL). After 5 minutes, 20% sodium carbonate (2.0 mL) was added to the mixture. After being kept in total darkness for 1 h, the absorbance was measured at 750 nm using a spectrophotometer (U-2001, Hitachi Instruments Inc., Tokyo, Japan). Amounts of TP were calculated using a gallic acid calibration curve. The results were expressed as gallic acid equivalents (GAE) g/g of dry plant matter [3].

### 3.4. Determination of Total Flavonoid Contents (TF)

The TF were measured following a previously reported spectrophotometric method [50]. Briefly, extracts of each plant material (1 mL containing 0.1 mg/mL) were diluted with 4 mL water in a 10 mL volumetric flask. Initially, 5% NaNO_2_ solution (0.3 mL) was added to each volumetric flask; at 5 min, 10% AlCl_3_ (w/w) was added; and at 6 min, 1.0 M NaOH (2 mL) was added. Water (2.4 mL) was then added to the reaction flask and mixed well. Absorbance of the reaction mixture was read at 430 nm. The results were expressed in mg quercetin/g of dry plant matter by comparison with the quercetin standard curve, which was made in the same condition.

### 3.5. Determination of Antioxidant Activities

#### 3.5.1. DPPH Radical Scavenging Assay

1,1-Diphenyl-2-picryl-hydrazyl (DPPH) was purchased from Sigma–Aldrich (USA). Butylated hydroxytoluene (BHT) and α-tocopherol were purchased from Merck (India). In order to determine the radical scavenging ability, the method reported by Mensor *et al.* [51], was used. Briefly, 1 mL alcohol solution of DPPH was added to 2.5 mL samples containing different concentrations, originating from different parts of ginger varieties’ extracts. The samples were first kept in a dark place at room temperature and their absorbance was read at 518 nm after 30 min. The antiradical activity (AA) was determined using the following formula:

AA%=100-((Abs:sample-Abs:empty sample)×100)/Abs:control

Blank samples contained 1 mL ethanol + 2.5 mL from various concentrations of ginger extract; control sample containing 1 mL of 0.3 mM DPPH + 2.5 mL ethanol. The concentration of the samples, the control and the empty samples were measured in comparison with ethanol. One synthetic antioxidant, BHT (butyl hydroxyl toluene) and α-tocopherol, were used as positive controls.

#### 3.5.2. Reducing Ability (FRAP Assay)

The determination of the total antioxidant activity using FRAP assay in the extract followed a modified method reported by Benzie *et al.* [52]. The stock solution included 300 mM acetate buffer (3.1 g C_2_H_3_NaO_2_·3H_2_O and 16 mL C_2_H_4_O_2_) at pH 3.6, 10 mM TPTZ (2,4,6-tripyridyl-*s*-triazine) solution in 40 mM HCl, and 20 mM FeCl_3_·6H_2_O solution in distilled water. Then acetate buffer (25 mL) and TPTZ (2.5 mL) were mixed together with FeCl_3_·6H_2_O (2.5 mL). The temperature of the solution was raised to 37 °C before it was used. Plant extracts (100 mg/mL) were allowed to react with the FRAP solution (2.85 mL) for 30 min under dark conditions. The absorbance was measured at 593 nm. The standard curve was linear between 200 and 1,000 μM FeSO_4_. Results were expressed in μM Fe(II)/g of dry plant matter and compared with those of standards for BHT, ascorbic acid, and α-tocopherol.

### 3.6. Statistical Analysis

The experimental design was factorial based on randomize complete block design (RCBD) results were expressed as mean ± standard deviation of three replicates. Where applicable, the data were subjected to one-way analysis of variance (ANOVA) and the differences between samples were determined by Duncan’s Multiple Range test using the Statistical Analysis System (SAS, 1999) and MSTATC programmes. P-value of <0.05 was regarded as significant.

## 4. Conclusions

This paper describes the impact of imposing varying levels of glasshouse irradiance on the accumulation and distribution of TP and TF content. It was deduced that Halia Bentong and Halia Bara contain substantial amounts of TF under 310 μmol m^−2^s^−1^ of sun irradiance. Varying the level of glasshouse irradiance from 790 to 460 μmol m^−2^s^−1^ led to a further increase in the accumulation of TF and favored greater partitioning to the leaves. The latter was future enhanced when the shade level continued to increase to 310 μmol m^−2^s^−1^. More information on TP and TF production under different light intensities would help us to establish a greater degree of accuracy on this matter. The results also showed good potential for phenolic compounds, especially flavonoids, in ginger leaves with high antioxidant activities. Thus, it seems that leaves of young ginger could be used for traditional medicine and food flavoring.

## Figures and Tables

**Table 1:** Accumulation and partitioning of TF and TP in different plant parts of two varieties of Z*ingiber officinale* grown under different light intensities.

Light Intensities (μmol m^−2^s^−1^)	Plant Part	TF (mg Quercetin/g dry weight)		TP (mg Gallic acid/g dry weight)	

		Halia Bentong	Halia Bara	Halia Bentong	Halia Bara
310	Leaves	5.95 + 0.2^c^	8.45 ± 0.38^a^	27.43 ± 2.34^e^	31.73 ± 2.10^cd^
	Stems	1.83 + 0.22^hi^	1.96 ± 0.28^h^	6.38 ± 1.25^h^	7.11 ± 1.58^gh^
	Rhizomes	3.91 + 0.083^efg^	4.34 ± 0.08^e^	8.9 ± 0.23^fgh^	9.48 ± 0.21^fgh^

460	Leaves	5.04 + 0.27^d^	5.7 ± 0.09^cd^	28.96 ± 1.55^de^	34.16 ± 4.8^bc^
	Stems	1.27 + 0.2^i^	1.47 ± 0.21^hi^	7.33 ± 1.13^fgh^	8.432 ± 1.19^fgh^
	Rhizomes	3.47 + 0.14^fg^	4.03 ± 0.061^efg^	9.69 ± 0.38^fgh^	11.22 ± 0.16^fg^

630	Leaves	4.14 + 0.18^ef^	6.12 ± 0.015^c^	31.1 ± 0.98^cde^	37.33 ± 4.45^ab^
	Stems	1.3 + 0.24^hi^	1.55 ± 0.33^hi^	7.47 ± 1.37^fgh^	8.83 ± 1.82^fgh^
	Rhizomes	3.37 + 0.079^g^	3.97 ± 0.28^efg^	9.81 ± 0.21^fgh^	11.05 ± 0.77^fg^

790*	Leaves	5.71 + 0.54^cd^	7.05 ± 1.67^b^	33 ± 1.13^cd^	39.06 ± 9.23^a^
	Stems	1.26 + 0.12^hi^	1.5 ± 0.14^hi^	7.8 ± 0.68^fgh^	8.56 ± 0.81^fgh^
	Rhizomes	3.66 + 0.125^fg^	4.14 ± 0.13^ef^	10.22 ± 0.33^fgh^	11.53 ± 0.36^f^

All analyses are the mean of triplicate measurements ± standard deviation. Means not sharing a common letter were significantly different at P ≤ 0.05.

*: See reference [12].

**Table 2:** DPPH scavenging activities of the methanol extracts (45 μg/mL) from different plant parts of two varieties of *Zingiber officinale.* BHT and α-tocopherol were used as positive controls.

Light Intensities (μmol m^−2^s^−1^)	Extraction Source	Halia Bentong	Halia Bara
310	Leaves	59.02 ± 0.87^b^	65.26 ± 0.9^a^
	Stems	30.31 ± 1.84^hi^	29.59 ± 0.59^i^
	Rhizomes	41.36 ± 0.63^f^	47.26 ± 0.92^e^

790*	Leaves	51.12 ± 1.65^d^	56.36 ± 0.97^c^
	Stems	32.85 ± 0.57^g^	31.45 ± 1.49^gh^
	Rhizomes	51.41 ± 0.51^d^	58.22 ± 1.19^b^

All analyses were mean of triplicate measurements ± standard deviation. Results expressed in percent of free radical inhibition. Means not sharing a common letter were significantly different at P ≤ 0.05.

*: See reference [12].

**Table 3:** FRAP activity in different parts of two varieties of *Zingiber officinale* grown under different light intensities. BHT, α-tocopherol and vitamin C were used as positive controls.

Light Intensities (μmol m^−2^s^−1^)	Extraction Source	Halia Bentong	Halia Bara
310	Leaves	552.24 ± 32.4^f^	587.31 ± 25.6^e^
	Stems	378.4 ± 48.2^h^	372.33 ± 32.33^hi^
	Rhizomes	692.71 ± 16.48^c^	788.57 ± 22.6^a^

790	Leaves	541.55 ± 34.1^g^	574.9 ± 58.14^e^
	Stems	381.11 ± 48.7^h^	363.1 ± 21.43^i^
	Rhizomes	677.2 ± 18.38^d^	770.4 ± 43.11^b^

All analyses were the mean of triplicate measurements ± standard deviation. Results are expressed in μmol Fe(II)/g dry weight. Means not sharing a common letter were significantly different at P ≤ 0.05.
